# A single anion exchange chromatography step enables scalable, high-yield purification of hepatitis B virus capsid virus-like particles

**DOI:** 10.3389/fmicb.2026.1904236

**Published:** 2026-10-09

**Authors:** A. Seidler, S. Ambike, C. Peters, R. Bester, M. Sattler, A. Geerlof, U. Protzer

**Affiliations:** 1 Institute of Virology, School of Medicine, Technical University of Munich and Helmholtz Munich, Munich, Germany; 2 Program in Emerging Infectious Diseases, Duke-NUS Medical School, Singapore, Singapore; 3 Electron Microscopy Faculty, Chemistry Department, Technical University of Munich, Munich, Germany; 4 Hemholtz Munich, Institute of Structural Biology, Munich, Germany; 5 German Center for Infection Research (DZIF), Munich Partner Site, Munich, Germany

**Keywords:** anion exchange chromatography, capsid, HBV, purification, VLP

## Abstract

**Introduction:**

Hepatitis B Virus (HBV) causes a significant global health burden. The HBV core (HBc) protein forms the HBV capsid and has a crucial role in the virus replication cycle. When expressed across different platforms, HBc can self-assemble into HBV-capsid-based virus-like particles (VLPs), with a wide range of applications in both fundamental and pharmaceutical research. Due to their ability to display or crosslink foreign antigens on the surface, HBc VLPs are used as vaccine antigens. However, conventional VLP purification methods face challenges in scalability and achieving high yields. Therefore, investigating the generation and purification process of these VLPs is of high interest.

**Methods:**

HBc VLPs composed of three different forms of HBc protein were expressed in *E. coli* – D (full-length, 183 aa), ΔD_163_ (C-terminal partially truncated, 163 aa), and ΔD_149_ (C-terminal fully truncated, 149 aa). Polyethyleneimine precipitation (PEI) was performed for excess nucleic acid reduction, followed by two stages of ammonium sulfate precipitation (AS). For a final purification anion-exchange chromatography (AEX) was performed. Purified HBc VPLs were analyzed for purity and stability by SDS-PAGE, native agarose gel electrophoresis, ELISA, Dynamic light scattering, electron microscopy and host cell protein and DNA and endotoxin assays.

**Results:**

The single AEX step method resulted in high purity (>85%) and reduced host-cell residues comparable to those detected in a sample produced under good manufacturing practice (GMP) conditions. The HBc VLP variants yielded 5.2 mg, 25.9 mg, and 27.8 mg purified capsids per liter E. coli culture, respectively. Our data showed that all three VLP forms preserved their correct assembly and exhibited minimal aggregation.

**Discussion:**

We successfully designed an HBc-VLP purification technique for the purification of different HBV core antigen particles. The purification resulted in highly pure and little aggregated VLP. This purification method is therefore well-positioned to accelerate studies of HBc VLPs.

## 1 Introduction

Chronic hepatitis B, caused by the hepatitis B virus (HBV), is a major global health concern. It affects more than 240 million individuals worldwide and resulted in approximately 1.1 million deaths in 2024 (WHO, 2026). Current standard treatments using nucleos(t)ide analogs rarely achieve a functional cure in infected individuals (Gehring and Protzer, 2019). The covalently closed circular DNA (cccDNA), the persistent form of HBV in the nucleus of an infected cell, poses a daunting challenge, making it difficult to cure the infection with current therapeutic strategies. Failure to eliminate cccDNA may result in viral reactivation upon cessation of medication (Lucifora and Protzer, 2016; Revill et al., 2016). This requires long-term treatment, underscoring the critical need for novel, curative therapeutic strategies.

The HBV core protein (HBc) is the only component of the HBV capsid and plays a pivotal role at various stages of viral replication (Zlotnick et al., 2015). The viral capsid encapsidates the pregenomic RNA (pgRNA) of HBV and serves as the site for reverse transcription and formation of the HBV DNA genome (Shen et al., 2023; Tan et al., 2015). The HBc monomer is a 21 kDa protein composed of 183 amino acids, with the first 149 aa forming the assembly domain and aa 150 to 183 constituting the arginine-rich C-terminal domain (CTD) responsible for nucleic acid binding (Birnbaum and Nassal, 1990). Following their dimerization, HBc dimers can spontaneously self-assemble into icosahedral capsids of either T = 3 or T = 4 symmetry, comprising 90 or 120 dimers, respectively (Alom et al., 2024; Selzer and Zlotnick, 2015). Truncations in the CTD up to residue 149 have been reported to enhance bacterial expression levels and result in a predominance of T = 4 capsids, without causing any noticeable alterations in particle morphology (Wang et al., 2019). This adaptability renders recombinant HBV capsids an important platform for investigating viral assembly mechanisms but also enables the development of virus-like particles (VLPs) for diverse biomedical applications.

Prior reports have demonstrated that HBc-based VLPs expressed in bacterial systems are well suited for a range of biological applications (Hassebroek et al., 2023; Petrovskis et al., 2021). Leveraging their immunogenic properties, HBc VLPs have been used to develop therapeutic vaccines for chronic hepatitis B (Backes et al., 2016; Buchmann et al., 2013; Su et al., 2024). Owing to their unique structural characteristics and ability to self-assemble, HBc VLPs have also emerged as a versatile platform for presenting foreign antigens, expanding their use as a vaccine platform (Dishlers et al., 2023; Fatema et al., 2025; Moradi Vahdat et al., 2021). Beyond these applications, HBc VLPs can also be engineered for the targeted delivery of therapeutic agents or adjuvants (Mohamed Suffian et al., 2017; Sacherl et al., 2023). Additionally, purified HBc VLPs are valuable for screening antiviral compounds, such as capsid assembly modulators (Burdette et al., 2023), and for structural and mechanistic studies (Lecoq et al., 2023) aimed at an in-depth understanding of virion assembly and HBV pathogenesis. Given such diverse applications, the need for more efficient production methods and rapid purification protocols to obtain highly pure HBV capsid particle variants is steadily growing.

A widely used conventional method at the laboratory level for purifying HBc VLPs is sucrose density gradient centrifugation (Chevreuil et al., 2020; Fatema et al., 2025; Zhang et al., 2023). Additionally, various chromatography techniques, such as ion exchange chromatography and size-exclusion chromatography (SEC), have been employed to purify HBc VLPs (Berke et al., 2017; Petrovskis et al., 2021; Selzer et al., 2014). However, these methods often require combining multiple approaches and several steps to achieve high purity. On the other hand, more selective methods, such as affinity chromatography, can achieve high purity; however, they often yield lower amounts and may require the addition of a tag. This can limit their applicability and potentially interfere with downstream applications (Bin Mohamed Suffian et al., 2017; Zhang et al., 2023). As previously reported, two consecutive SEC steps are commonly used to purify HBc VLPs, resulting in broad elution peaks that make separation of host cell proteins and the VLPs difficult (Orabi et al., 2015). Adapting the protocol to two anion-exchange chromatography (AEX) largely reduced the yield. As HBc VLPs are known to associate with host RNAs (Porterfield et al., 2010), we introduced a polyethyleneimine (PEI) precipitation step to remove excess surface-associated host nucleic acids that are not encapsulated within the capsids, thereby eliminating the need for a second AEX run. Here, we present a one-step ion-exchange chromatography method for purifying three variants of HBc VLPs. These variants were derived from C-terminal truncations of the HBV genotype D HBc protein, namely, the full-length HBc (D), semi-truncated HBc (ΔD_163_), and truncated HBc (ΔD_149_), with the latter exhibiting a complete absence of the nucleic acid-binding CTD. All three capsid variants were successfully purified to comparable purity levels.

## 2 Materials and methods

The pRSF plasmid backbone was kindly provided by Prof. Dr. Michael Nassal (University of Freiburg, Germany). All HBc variants (D residues 1–183, ΔD_163_ residues 1–163, ΔD_149_ residues 1–149) are of genotype D, subtype ayw and were synthesized by GeneArt gene synthesis (ThermoFisher, USA) after codon optimization for expression in *E. coli*. The respective HBc genes were cloned downstream of the T7 promoter by conventional cloning using *Pfo*I and *BshT*I restriction sites in the pRSF backbone. The resulting plasmid constructs were sequenced using whole-plasmid sequencing (Eurofins, Luxembourg) to verify the correctness of both the backbone and the inserted sequences.

### 2.1 Expression of HBV capsid particles

*E. coli* BL21 (DE3) containing the respective pRSF-1b expression plasmids were pre-cultured overnight at 37 °C, constantly shaking at 160 rpm in lysogeny broth (LB) supplemented with kanamycin. On the following day, the pre-culture was added to 1 L terrific broth (TB) medium (Krause et al., 2010) (ThermoFisher, USA) to achieve a starting optical density (OD_600nm_) of 0.2 of the expression cultures. The cultures were allowed to grow until an OD_600nm_ of 0.8–1.0 was reached. The cultures were then induced with 1 mM isopropyl-1-thio-β-d-galactopyranoside (IPTG; Sigma-Aldrich, USA) overnight for 16 h. The expression cultures were grown at 25 °C with constant shaking at 160 rpm throughout the expression process. After expression, the bacteria were harvested by centrifuging at 5000 rpm for 10 min at 4 °C. The pellets were resuspended in 40 mL lysis buffer (50 mM Tris–HCl pH 8.0, 1 M NaCl, 10 mM MgCl2, 1 mM AEBSF, HCl) supplemented with 10 μg/mL DNase I. Cell suspension was sonicated 2 × 3 min with 30 s pause in between, at 30% on and 60% amplitude on ice-water using a VS70T probe on a Bandelin Sonopuls HD2200 to ensure complete bacterial lysis. Bacterial debris was removed by centrifugation at 20,000 rpm for 1 h at 4 °C.

### 2.2 Protein precipitation using PEI and ammonium sulfate

A 5% (v/v) stock solution of branched polyethyleneimine (PEI) with an average 60,000 M_W_ (Cat. No. 33141.03, Serva, Germany) in 0.5 M Tris buffer (pH 8.0) was added to the supernatant at 4 °C while stirring vigorously to a final concentration of 0.5% (v/v). The mixture was incubated for 30 min at 4 °C, followed by centrifugation (20 min at 25,000 rpm). The supernatant was retained for ammonium sulfate (AS) precipitation.

The first AS precipitation was performed at 90% (w/v) saturation at 4 °C. A defined amount of AS (Roth, Germany) was added stepwise to the supernatant while stirring continuously, and the mixture was incubated for 1 h. The solution was centrifuged (30 min at 25,000 rpm), and the resulting pellet was dissolved in Tris-Buffered Saline (TBS; 50 mM Tris–HCl, pH 7.5, 150 mM NaCl) to match the volume of the discarded supernatant. Remaining solid contaminants were removed by centrifugation (20 min at 25,000 rpm), and the supernatant was dialyzed overnight against 2 L of TBS using a 10 kDa cutoff dialysis membrane (Serva, Germany). The contents of the dialysis tube were centrifuged (20 min at 25,000 rpm) and the resulting supernatant subjected to subsequent AS precipitation.

The second AS precipitation was carried out at either 40% (w/v) saturation for capsids composed of full-length HBc or 25% (w/v) saturation for the two truncated variants. AS was added stepwise under continuous stirring and incubated for 1 h, followed by centrifugation (30 min at 25,000 rpm). The resulting pellet was resuspended in AEX buffer A (10 mM MES/Tris, pH 6.0) in a volume equivalent to that of the discarded supernatant. After centrifugation (20 min at 25,000 rpm), the supernatant was filtered through a 0.45 μm filter and kept free of air bubbles until further processing. All the centrifugation steps with protein solutions were carried out at 4 °C using a A27-8×50 rotor in a Sorvall Lynx 6,000 centrifuge (Thermo Scientific).

### 2.3 Anion exchange chromatography (AEX)

Anion exchange chromatography was performed at room temperature using a 5 mL HiTrap Q HP column (Cytiva, USA) packed with Q Sepharose™ High Performance strong anion exchange resin consisting of quaternary ammonium groups coupled to a cross-linked agarose matrix, with a particle size of approximately 34 μm. The column was connected to the HPLC system (Äkta Purifier 10, Cytiva). The column containing quaternary ammonium groups immobilized on a cross-linked agarose matrix (average particle size approximately 34 μm) functions as a strong anion exchanger. The column was pre-equilibrated with AEX buffer A containing 5% (v/v) AEX buffer B (10 mM MES/Tris, pH 6.0, 1.0 M NaCl). After the sample application, the column was washed with AEX buffer A containing 5% (v/v) AEX buffer B (50 mM NaCl), followed by a linear gradient of 1 column volume (CV) from 5 to 20% (v/v) AEX buffer B (50–200 mM NaCl). Bound HBc proteins were eluted using a linear gradient (10 CVs) from 20 to 80% (v/v) AEX buffer B (200–800 mM NaCl). Fractions were collected at defined intervals and monitored by photodiode-array detectors at 254 and 280 nm. Fractions containing HBc proteins with an A_280_ above 0.2 and the desired ratio between A_260_ and A_280_ (determined using a Peqlab Nanodrop 2000 spectrophotometer, Thermo Scientific) (for HBV D = 1.60–1.70; ∆D_163_ = 1.10–1.20; ∆D_149_ = 0.65–0.70), were pooled and subsequently dialyzed against 2 L of storage buffer (PBS containing 6 mM EDTA) to remove excess salts and buffer components. The total soluble protein amounts were determined by the Bradford assay (Bradford, 1976). The purified capsid particles were then stored at 4 °C throughout the study.

### 2.4 Determination of host cell contaminations

Residual *E. coli* proteins and endotoxin levels in the purified preparation were quantified using the *E. coli* HCP ELISA kit (Cygnus, USA); limit of detection (LOD) ~ 0.4 ng/mL, lower limit of quantification (LLOQ) ~ 3 ng/mL and Pierce™ Chromogenic Endotoxin Quant Kit (Thermo Fisher, USA; LOD and LLOQ 0.01 EU/ml), respectively, following the manufacturer’s instructions. For host cell DNA contamination assessment, total DNA was first extracted from the samples using the NucleoSpin Tissue kit (Macherey-Nagel, Germany). Quantitative PCR (qPCR) was then performed on the extracted DNA using the LightCycler 480 system and SYBR™ Green PCR Master Mix, with ER-F3 (GCTCGTGTTGTGAAATGTTG) and ER-R3 (GTAAGGGCCATGATGACTTG) as forward and reverse primers, respectively (Lee et al., 2010).

### 2.5 Sodium dodecyl sulfate-polyacrylamide gel electrophoresis (SDS-PAGE)

The samples collected during and after the purification process were analyzed by SDS-PAGE using 15% polyacrylamide gels. 10 μL of purified protein samples were denatured by mixing with 2 μL Laemmli SDS buffer, reducing (6x; ThermoFisher, USA), followed by heating at 95 °C for 10 min Samples were then centrifuged briefly at 15,000 rpm for 1 min before loading onto the gel for electrophoresis. SDS-PAGE was conducted with the Mini-PROTEAN Tetra Vertical Electrophoresis Cell (Bio-Rad, USA) system under a constant current of 20 mA per gel. Following electrophoresis, the gels were stained using Coomassie Brilliant Blue R-250 (Roth, Germany) and analyzed using ChemiDoc MP (Bio-Rad, USA) as per the manufacturer’s instructions. The purity of the final product was assessed using Image Lab 6.1 software (Bio-Rad, USA).

### 2.6 Native agarose gel electrophoresis (NAGE)

Native agarose gel electrophoresis was used to analyze capsids in their native form. A 1% agarose (Sigma-Aldrich, USA) gel was prepared in 1x Tris-Acetate Ethylenediaminetetraacetic acid (EDTA; TAE) buffer (40 mM Tris, 5 mM acetate [CH_3_COONa], and 0.9 mM EDTA, pH 7.9) using the PerfectBlue Mini M Gel System (VWR, USA). Electrophoresis was performed at 110 V for 1 h. Following electrophoresis, gels were sequentially stained with Roti®GelStain (Roth, Germany) to detect encapsulated nucleic acids and Coomassie Brilliant Blue R-250 (Roth, Germany) to visualize native capsid protein bands. Gel imaging and analysis were performed using the ChemiDoc MP system (Bio-Rad, USA).

### 2.7 Western blot analysis

Western blot analysis was performed following the wet transfer of proteins from polyacrylamide gels or agarose gels onto Amersham™ HybondTM PVDF membrane (0.45 μm; GE Healthcare, USA) using Mini Trans-Blot® Cell (Bio-Rad, USA). Membrane blocking was carried out for 1 h using 5% (w/v) skim milk powder (Roth, Germany) in 1 × Tris-buffered saline (TBS) supplemented with Tween 20 (TBS-T) buffer (20 mM Tris, 150 mM NaCl, and 0.1% Tween 20, pH 7.6). The primary antibody 8C9-11 (anti-HBc monoclonal antibody, Core Facility Monoclonal Antibodies, Helmholtz Munich) (Ko et al., 2018), diluted 1:1 in blocking buffer, was applied overnight at 4 °C under constant mixing. Membranes were washed three times with TBS-T (50 mM Tris–HCl, pH 7.6, 150 mM NaCl, 0.1% Tween 20) before incubation with the HRP-conjugated secondary antibody (goat anti-mouse, Sigma-Aldrich, USA). After additional washing, signal detection was performed using an enhanced chemoluminescence (ECL) substrate (Thermo Fisher, USA) and analyzed with the ChemiDoc MP system (Bio-Rad, USA).

### 2.8 In-house sandwich HBcAg enzyme-linked immunosorbent assay (ELISA)

The integrity of HBc VLPs was assessed using an in-house ELISA. Microtiter plates were coated with 1 μg/mL anti-HBcAg capture antibody overnight at 4 °*C. prior* to adding the protein samples, the wells were blocked with 5% BSA (Sigma-Aldrich, USA) in phosphate-buffered saline (PBS) for 1 h at room temperature to minimize unspecific binding of the detection antibody. Thereafter, purified protein samples (20 ng/mL) were added to the wells along with a dilution series of BSA standard (ranging from 0 to 50 ng/mL). The samples were prepared in assay diluent (1% BSA/PBS) and incubated for 2 h at room temperature. The captured HBc VLPs were detected by the detection antibody after 1 h of incubation. Both antibodies were kindly provided by the Centro de Ingeniería Genética y Biotecnología de Cuba. Color development was achieved with TMB Single Solution (Life Technologies, USA), and the reaction was stopped by adding 2 M sulfuric acid (Roth, Germany). Absorbance was recorded at 450 nm with a reference wavelength of 560 nm using an Infinite F200 plate reader. Each procedural step was followed by washing with 0.05% PBS-T as indicated.

### 2.9 Dynamic light scattering (DLS)

To determine the particle size distribution, DLS was performed on a Zetasizer Nano ZS instrument (Malvern Instruments Ltd., Malvern, UK). 5 μg of HBc VLPs were diluted in 10 mM NaCl and analyzed. The DLS results were analyzed using the DTS software (Malvern, version 6.32).

### 2.10 Transmission electron microscopy (TEM)

HBc VLPs were observed under a transmission electron microscope. Continuous carbon grids were glow-discharged for 30 s at 20 mA using GloQube Plus (Quorum, UK). Sample absorption was performed for 1 min before the samples were stained with 2% uranyl acetate solution for 30s. TEM was conducted using JEM-1400Plus (JEOL, Japan) operated at 120 kV, equipped with CCD camera Ruby (JEOL, Japan). Micrographs were taken at a nominal magnification of 60 k with a final pixel size of 2.75 A/px.

## 3 Results

### 3.1 Three different variants of HBc monomers can be efficiently expressed in an inducible bacterial system

VLPs made of varying lengths of HBc were successfully synthesized using pRSF constructs harboring the HBc gene under an inducible T7 promoter. Proteins were expressed in *E. coli* BL21 (DE3) cells carrying the respective plasmid. To minimize the risk of protein aggregation and to favor protein synthesis and VLP assembly, the expression cultures in TB medium were maintained at lower temperatures than the bacteria’s optimal growth temperature (Asor et al., 2020; Hilmer et al., 2008). The expression cultures were initiated at an OD_600nm_ of 0.2 and reached an OD_600nm_ of approx. 1.0 within 4–5 h of growth at 25 °C. At this point, protein expression was induced by adding 1 mM IPTG. Following an overnight incubation period of approximately 16 h, the OD_600nm_ of the cultures was measured at just above 3.0, with pellet weights reported in Table 1.

**Table 1:** Final density of *E. coli* expression cultures, protein yield, and product recovery Yield measured by Bradford assay.

Sample	OD_600nm_ after expression	Bacterial pellet weight [g]	OD/g wet pellet	Yield per l culture [mg]	Product recovery [mg/kOD]
D	3.06	5.74	533	5.2	1.70
ΔD_163_	3.2	5.88	544	25.9	8.09
ΔD_149_	3.12	5.97	523	27.8	8.91

The SDS-PAGE analysis of bacterial lysate clearly showed bands corresponding to the expected molecular weights of the monomers of all three HBc variants upon overexpression. As shown in Figure 1A, the monomer of the full-length variant was observed at approximately 21 kDa, followed by the ΔD_163_ monomer at around 18 kDa and the ΔD_149_ variant at 15 kDa. The results were then confirmed by WB analysis using the in-house 8C9-11 antibody, which recognizes an N-terminal epitope of HBc. As expected, the WB analysis also showed well-resolved bands for the three HBc variants, with the same size as those observed on SDS-PAGE (Figure 1B).

**Figure 1:**
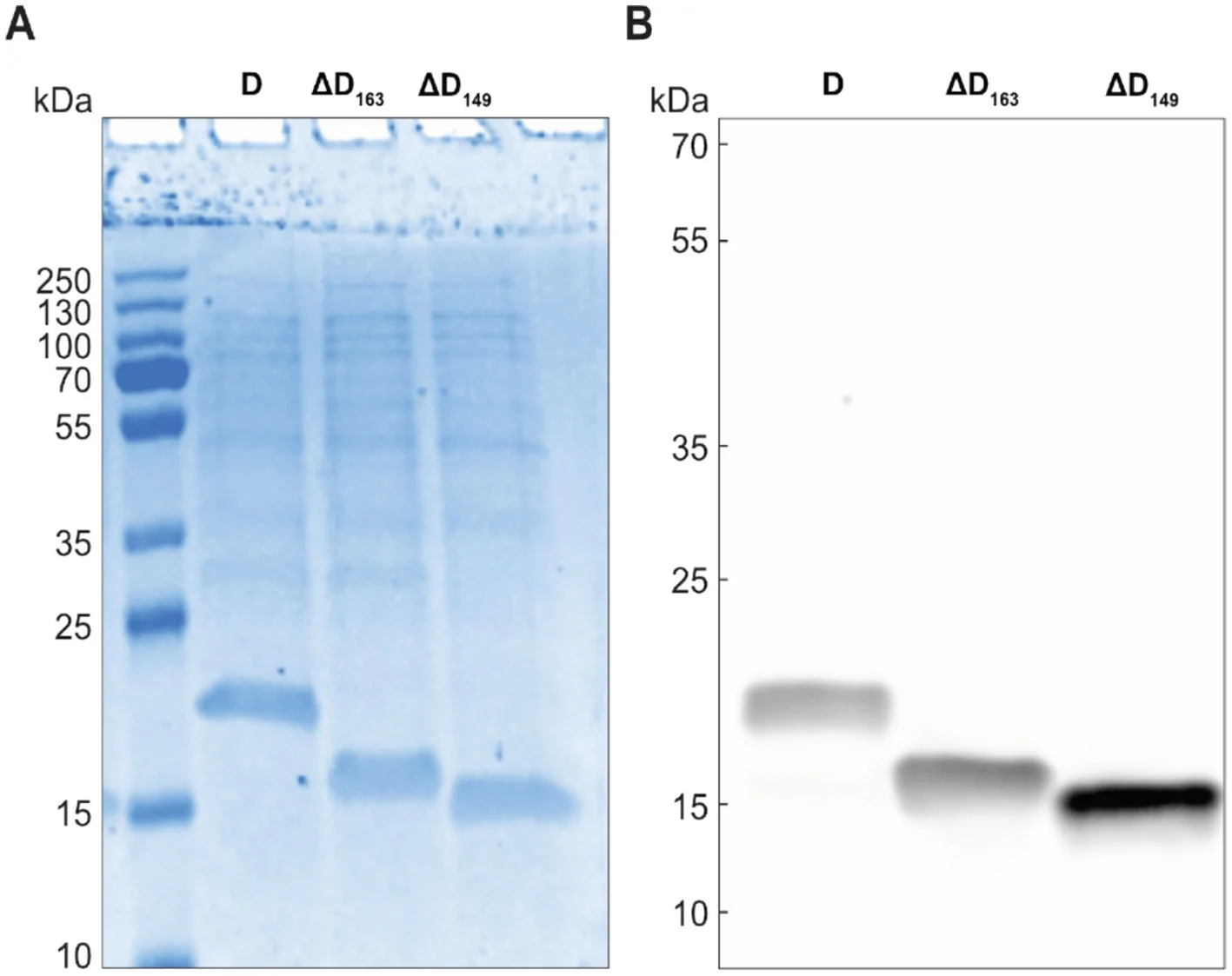
Expression of HBc variants in *E. coli*. **(A)** Bacterial cell lysates applied to SDS-PAGE and stained with Coomassie Brilliant Blue R-250 (10 μL sample) and **(B)** WB (5 μL sample, right). Staining of the membranes was done using anti-HBcAg antibody. Lanes: (1) PageRuler™ Plus Prestained Protein Ladder used as marker, (2) full-length HBc D (21 kDa), (3) semi-truncated HBc ΔD_163_ (18 kDa), (4) fully-truncated HBc ΔD_149_ (15 kDa).

### 3.2 An HBc VLP one-step anion exchange chromatography method produces highly pure and intact HBV capsid particles

Proteins expressed in bacterial systems often contain substantial levels of host-derived contaminants (Disela et al., 2024; İncir and Kaplan, 2024). The persistence of contaminating host cell nucleotides poses a major challenge for HBc purification, as the negatively charged nucleic acids in the lysate exhibit a strong affinity for anion-exchange resins. This competitive binding can reduce chromatographic resolution and lower final protein yield. To minimize host nucleic acid contamination, a DNase I treatment was performed during the lysis (Lim et al., 2018), followed by 0.5% (v/v) polyethyleneimine (PEI) precipitation of the supernatant after centrifugation of the lysate (Huber et al., 2019). Collectively, these steps reduced the risk of negatively charged nucleic acids binding to the chromatography column (Burgess, 2009; Stone et al., 2018) and substantially reduced the DNA content in the VLP preparations throughout the purification process.

Subsequently, an AS precipitation step (90% (w/v) saturation) was performed, followed by dialysis. This step was primarily aimed at removing the majority of the remaining PEI from the protein solution while retaining the maximum HBc content. To further improve the separation of HBc VLPs from host *E. coli* proteins, an additional AS precipitation was implemented. For VLPs formed out of truncated HBc proteins (ΔD_163_, ΔD_149_), a 25% saturation was applied, resulting in an almost complete precipitation of the HBc proteins. For VLPs formed out of full-length HBc (D) a 25% saturation only partially precipitated the protein. Therefore, the AS concentration was increased to 40%. This improved the yield of HBc proteins but also increased the amount of precipitated host cell proteins (Figures 2B,D,F).

**Figure 2:**
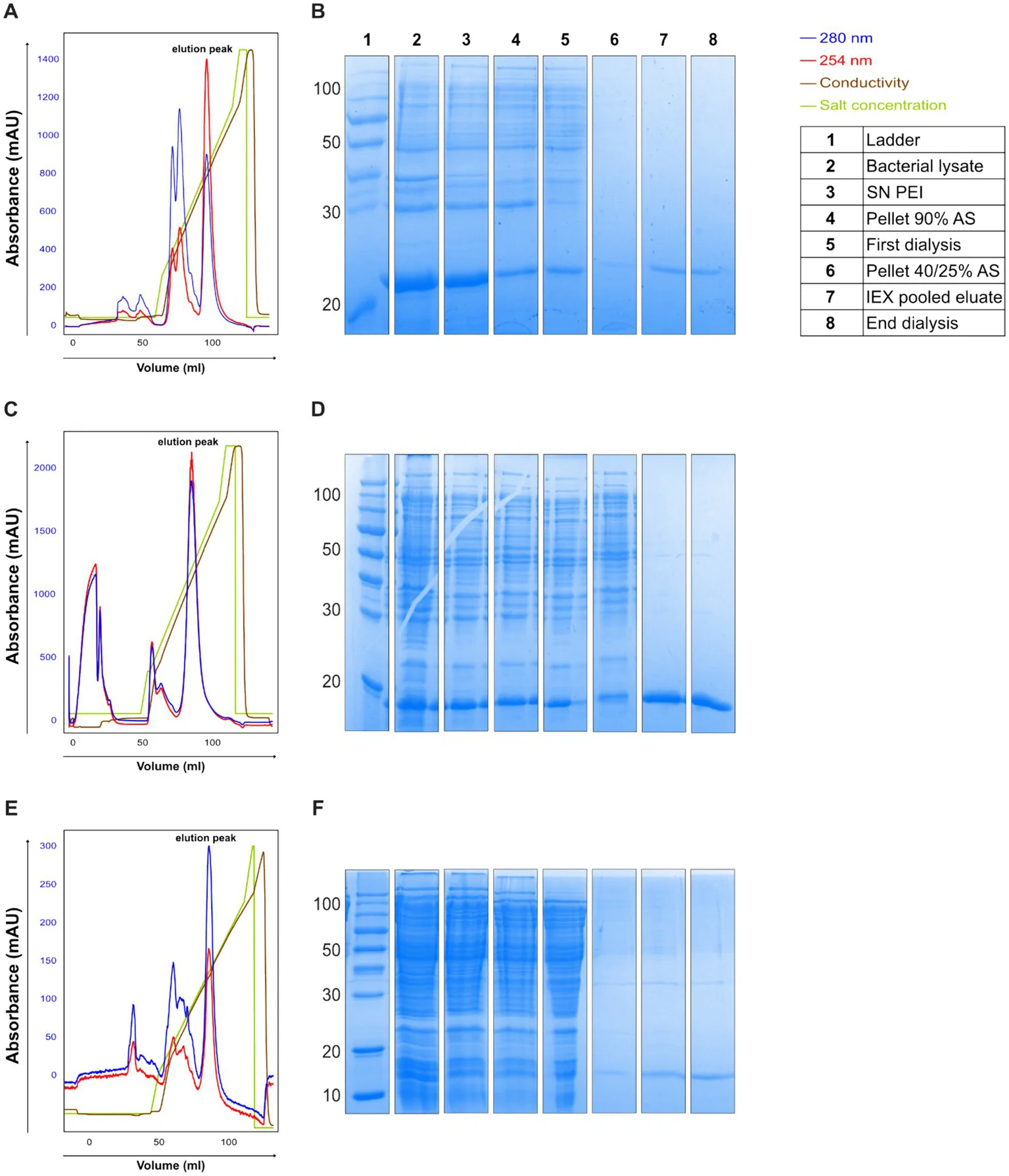
Representation of purification process HBcAg particles D, ΔD163, ΔD149. The chromatograms **(A,C,E)** show the characteristic peaks of the eluted HBc VLPs. SDS-PAGEs **(B,D,F)** show samples of the individual purification steps. 1 = ladder, 2 = bacterial lysate, 3 = PEI precipitation SN, 4 = 90% AS precipitation pellet, 5 = first dialysis, 6 = 40%/25% AS precipitation pellet, 7 = pooled eluates after AEX, 8 = end dialysis to final buffer (PBS, 6 mM EDTA, pH 7.4).

Resuspended pellets from the previous step were then subjected to anion exchange chromatography (AEX) using HiTrap Q HP column (Cytiva, USA) acting as a strong anion exchange resin. During the chromatography process (Figures 2A,C,E), the eluates were continuously monitored for the presence of soluble proteins and nucleic acids. Using the salt gradient, we were able to separate the capsids, as shown in Coomassie-stained gels (Figures 2B,D,F). For full-length D VLPs, the elution peak was defined by a high A_254 nm_ signal that exceeded the A_280 nm_ signal (average (av.) 1.67, Figure 2A). Although only the total nuclease content was measured by A_254 nm_, the findings were consistent with the presence of internally packaged nucleic acids, as shown later (Figure 3B). For ΔD_163_, the A_254 nm_ signal was only slightly higher than the A_280 nm_ signal (av. 1.15-fold), reflecting the reduced nucleic acid packaging capacity of these VLP (Figures 2B, 3B). In the case of ΔD_149_, which exhibits the lowest nucleic acid content among the three variants, the A_254 nm_ signal was notably lower compared to the A_280 nm_ signal (av. 0.67-fold) within the elution peak (Figures 2C, 3B). This could be due to the absence of the nucleic acid-binding CTD, indicating that the eluted particles primarily consisted of empty capsids, which might consequently result in a smaller margin within the A_254 nm_ peak.

**Figure 3:**
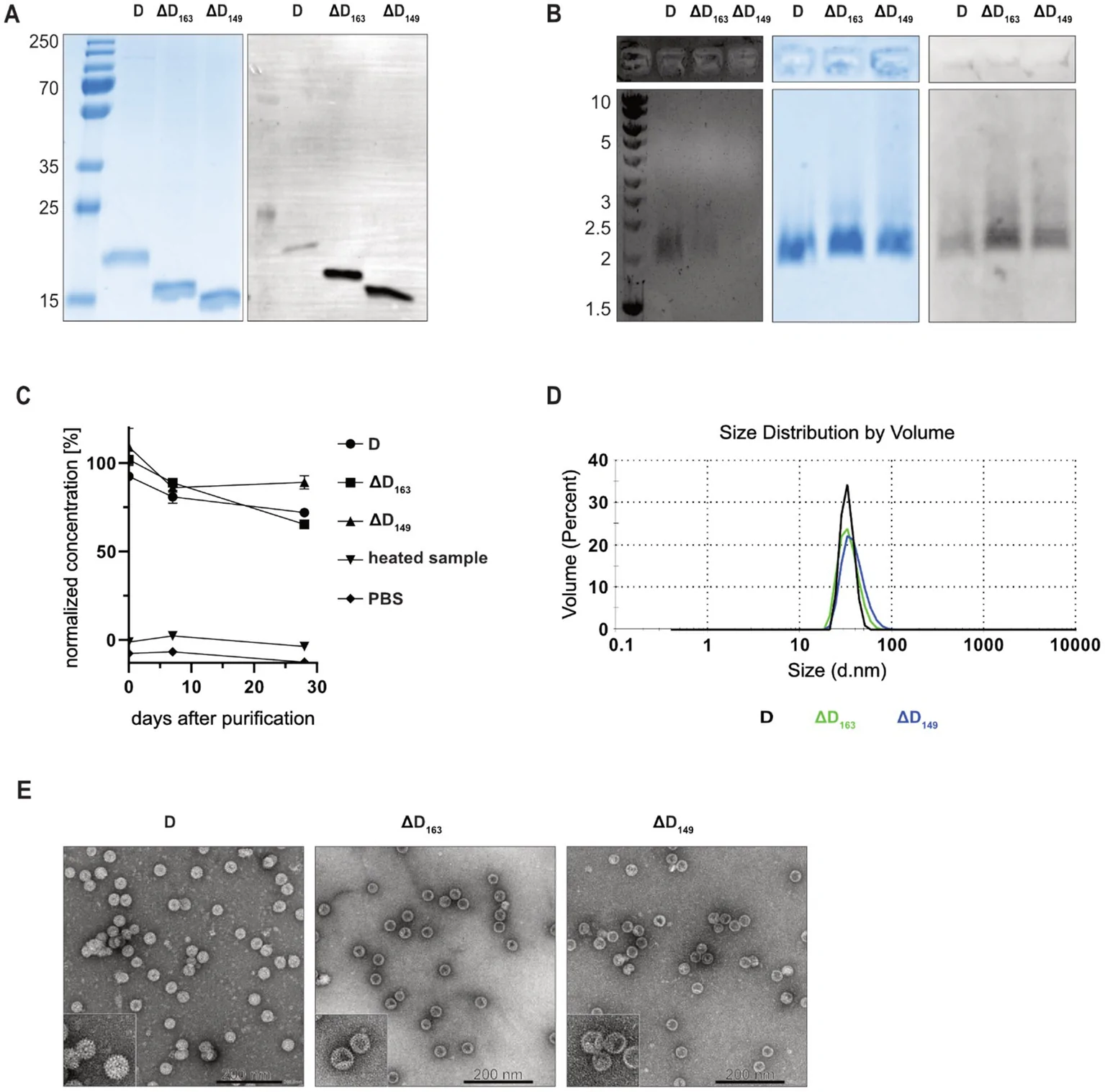
Verification of HBcAg particle presence after purification and stability assessment. **(A)** SDS-PAGE stained with Coomassie Brilliant Blue R-250 (left) and anti-HBc antibody (right); **(B)** NAGE stained with Roti®GelStain (left), Coomassie Brilliant Blue R-250 (mid) and anti-HBc antibody (right); **(C)** in-house HBcAg ELISA showing HBcAg particle stability over the course of 1 month (mean ± SD, *n* = 3); **(D)** DLS measurement showing size distribution by volume and **(E)** electron microscope images of HBcAg particles.

As expected, subsequent SDS-PAGE analysis of eluted fractions demonstrated markedly higher purity than samples collected after the second AS precipitation step. Notably, residual protein bands ranging from 30 to 150 kDa, observed in earlier steps, were markedly reduced (Figures 2B,D,F). Following a final dialysis step into the storage buffer (PBS, 6 mM EDTA), the concentration of the purified products was estimated using a Bradford assay, enabling yield calculations. The yield of D was lower than that of the other two variants, with recoveries of 1.70 mg/kOD for D, 8.09 mg/kOD for ΔD_163_, and 8.91 mg/kOD for ΔD_149_ (Table 1).

Finally, host cell contaminants in the purified samples were compared with an HBc VLP sample produced under good medical practice (GMP) conditions (Table 2). The GMP sample, produced by Eurofins (France), was similarly expressed in *E. coli* and then lysed, but AS precipitation was performed directly, omitting the prior PEI precipitation. Chromatography purification involved two steps: AEX and hydrophobic interaction chromatography (HIC). Values for host cell protein, host cell DNA, and endotoxin content were measured and calculated at a protein concentration of 0.1 mg/mL. Despite slightly higher values than in the GMP batch, all three HBc-VLP samples showed low levels of contaminants from bacterial proteins or toxins. Remarkably, an association between the HBc length and the presence of contaminants, along with the degree of purity, was observed. Here, HBc VLPs D showed the highest contamination levels, which decreased progressively as the HBc proteins were shortened. This suggested that the full-length HBc, in addition to encapsidating and binding more nucleic acids, has a greater tendency to bind *E. coli* proteins and possibly even endotoxin.

**Table 2:** Host cell protein, DNA, and endotoxin levels of capsid samples post purification.

Sample	Host cell Protein [ng/ml]	Endotoxin [EU/ml]	Host cell DNA [px/rnx]	Purity [%]
D	37.0 ± 21.9	78.1 ± 2.1	1.909 ± 0.357	85.9
ΔD_163_	1.2 ± 0.8	70.2 ± 0.4	0.232 ± 0.014	90.8
ΔD_149_	0.8 ± 0.1	59.5 ± 1.0	0.002 ± 0.002	94.3
GMP grade HBc VLP	<0.4	<0.01	43.600 ± 17.652	n.d.

Host cell protein, DNA, and endotoxin levels are normalized for 0.1 mg/ml protein (mean ± SD, *n* = 3).

Figure 4 provides a schematic overview of the final purification process established. To minimize PEI content and reduce host protein contamination in the purified product, two AS steps were performed after PEI precipitation (Dishlers et al., 2023; Petrovskis et al., 2021). This was followed by AEX to achieve a highly purified preparation of HBc VLPs. In summary, this streamlined approach focuses on optimizing the steps preceding AEX, providing an efficient and scalable method for purifying HBc VLPs in a single AEX run. The method ensures high purity across different capsid variants without the need for additional tags or multiple chromatography steps, resulting in higher protein yields and making it well-suited for downstream biological applications.

**Figure 4:**
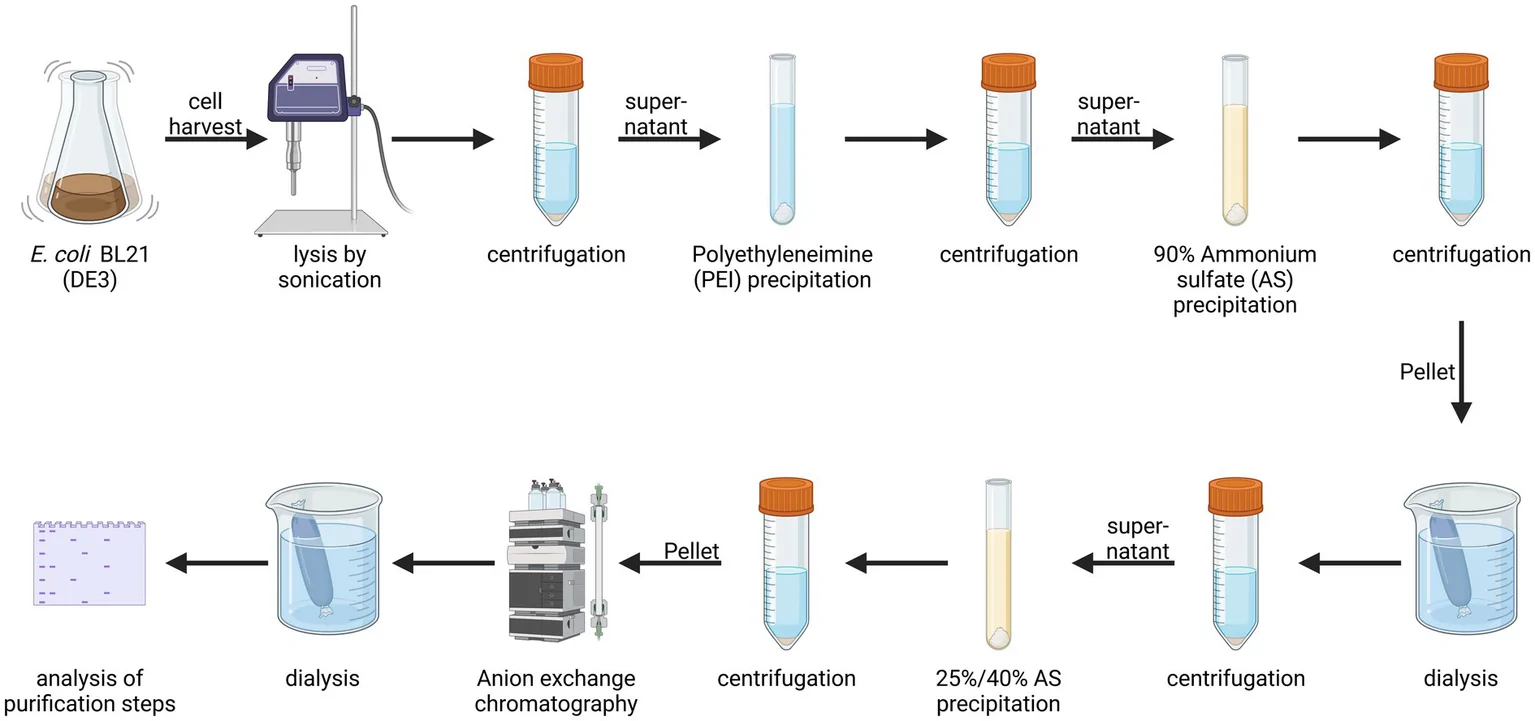
Schematic representation illustrating the HBcAg production and purification workflow.

### 3.3 HBV capsid particles retain integrity and exhibit minimal host cell residues after purification

The purified HBc VLPs were analyzed in detail for purity, integrity, and aggregate formation. The purity of the final products, assessed by SDS-PAGE (Figure 3A), exceeded 85% for VLP consisting of full-length HBc variant D and increased to over 90% for HBc ΔD_163_ and 94% for HBc ΔD_149_ (Table 2). A qualitative assessment of the native capsids by NAGE revealed that their assembled structure was retained after purification. The nucleic acid staining shows a strong band for full-length HBc D, while the bands become progressively weaker with HBc ΔD_163_ and HBc ΔD_149_, confirming the previously mentioned observation that HBc D VLPs are highly packed with nucleic acid, with progressively decreasing nucleic acid content with shortened C-termini in HBc VLPs ΔD_163_ and ΔD_149_. In contrast, the Coomassie-stained proteins showed dominant bands at positions corresponding to the size of the encapsulated RNA, as expected for recombinant VLPs (Heger-Stevic et al., 2018). The presence of clear bands corroborates the stability of the VLPs and their correct assembly (Figure 3B).

In the next steps, we assessed whether the integrity of the HBc VLP particles remained intact until 1 month after purification. VLP integrity was indicated by stable ELISA measurements (Figure 3C). Interestingly, the HBc VLPs from full-length HBc appeared to be slightly less stable compared to the particles consisting of truncated HBc (Figure 3C). In all three forms, however, the DLS measurement showed a single peak at around 30 nm, indicating uniform preservation of correctly assembled VLPs and corroborating the absence of particle aggregation (Figure 3D). TEM analysis confirmed well-distributed, homogenous capsids that do not form larger clusters in all three preparations. Interestingly, both larger T = 4 particles and the smaller T = 3 particles are contained in the final product, as shown in the microscopy images (Figure 3E).

## 4 Discussion

In this study, a method for the purification of HBc VLPs utilizing one AEX chromatography step was demonstrated. Using three different forms of HBc VLPs, we showed that alterations in HBc VLP’s RNA packaging properties, or its absence, influence the efficacy of the downstream purification method. We observed that a C-terminal truncation of HBc can enhance VLP yield and improve purification quality while preserving capsid assembly. Since not every application requires a full-length HBc (Dishlers et al., 2015; Sazegari et al., 2023; Schumacher et al., 2018), our protocol, depending on the specific downstream application, facilitates the efficient purification of HBc variants.

After successful expression of all three HBc variants, purification began with key precipitation steps. Purification largely preserved the internally packed RNA, as demonstrated by NAGE (Figure 3B (left)), but excess free nucleic acids were removed by PEI precipitation (Pérez-Vargas et al., 2014; Zhao et al., 2011). After the full purification process, only small amounts of residual host-cell DNA were detected, underscoring the effectiveness of PEI. Moreover, it should be noted that in previous protocols that lacked PEI precipitation, the AEX column rapidly overloaded, resulting in inefficient purification. The reason could lie in the negatively charged free nucleic acids binding to the positively charged column, which were now minimized by the precipitation step.

Subsequent AS precipitation (90% saturation) and the first dialysis into the AEX buffer were targeted to remove possible PEI residues. In the second AS precipitation step, separating VLPs from host cell proteins yielded the highest yield, with 25% saturation, for particles with truncated HBc proteins. For full-length HBc, we opted for 40% saturation to prevent loss of large quantities of the protein of interest, but at the cost of increased host-cell proteins in the purification preparation (Table 2). Nevertheless, a substantial portion of non-specific host cell proteins could be separated from the HBc VLPs.

During chromatography, the elution of HBc VLPs could be precisely monitored by their characteristic peaks. Due to the packed RNA inside the particles, the A_254 nm_ measurement (Figure 2A,C,E) shows a specific peak that even exceeds the peak of the A_280 nm_ measurement for full-length HBc VLPs and stays approximately equal for ΔD_163_ VLPs. A_254 nm_ signal remains low during the elution for ΔD_149_ VLPs, indicating the elution of mostly empty capsids (Valentic et al., 2022). Although these observations remain indirect, as total nucleic acids were monitored, they were later supported by nucleic acid staining in NAGE that showed co-localization with HBc VLP bands (Figure 3B).

Nevertheless, these findings highlight the distinct nucleic acid binding properties of the three HBc variants. Depending on the intended use of the VLP, this could be exploited during production, as it allows RNA-packed and almost empty particles to be selectively differentiated and sorted during chromatography. The yield of VLPs with truncated HBc was substantially higher than the VLP with full-length HBc containing arginin-rich domain. A similar trend has been observed previously. Factors such as mRNA confirmation and stability, as well as interactions between the C-terminal region of newly synthesized HBc and RNA are hypothesized to contribute to the limited expression of full-length variants (Berza et al., 2013; Sominskaya et al., 2013; Tan et al., 2003). The product recovery of full-length HBc VLPs further demonstrates the increased challenge of purifying the particles composed of full-length HBc by a one-step purification but simultaneously highlights the successful recovery of the particles with truncated HBc (Table 1). On the one hand, this results from the comparably higher loss of protein after each AS precipitation; on the other hand, more nucleic acids may adhere to the outside of full-length proteins or in the purification preparation, resulting in competitive binding to the column and reducing the chromatographic resolution (Valentic et al., 2022). Another reason could be that, in general, proteins adhered more prominently to VLPs containing full-length HBc than to those containing truncated HBc, as indicated by the measured host cell protein and endotoxin levels (Table 2). Overall, the yield of all three VLPs, combined with achieved purities of >85% for D, >90% for ΔD_163,_ and >94% for ΔD_149_ VLPs, is exceptional compared to similar one-step purification processes (Li et al., 2018; Lim et al., 2018).

After purification, host cell protein and endotoxin levels were slightly higher than in a GMP sample used for comparison, but remained low and majorly within the tolerated limits for medical applications, e.g., as vaccine antigens. Endotoxin levels were 78.1 ± 2.1 EU/ml, 70.2 ± 0.4 EU/ml, and 59.5 ± 1.0 EU/ml, normalized to a capsid concentration of 0.1 mg/mL, corresponding to 781 EU/mg, 702 EU/mg, and 595 EU/mg, respectively. Based on the K/M calculation defined in Ph. Eur. 5.1.10, these endotoxin levels would theoretically permit intravenous administration of up to 6.40 μg (D), 7.12 μg (ΔD_163_), and 8.40 μg (ΔD_149_) capsid per kg body mass, respectively (EDQM, 2023a, 2023b).

For residual host cell protein, no universal acceptance threshold has been established; acceptable levels are instead determined on a product-specific basis. A commonly used benchmark is <100 ng/mg product (100 ppm) (Graham et al., 2026). The measured HCP concentrations of 37.0 ± 21.9 ng/mL (D), 1.2 ± 0.8 ng/mL (ΔD_163_), and 0.8 ± 0.1 ng/mL (ΔD_149_), normalized to 0.1 mg/mL capsid, correspond to 370 ng/mg, 12 ng/mg, and 8 ng/mg capsid, respectively. Thus, the latter two variants were below the commonly used benchmark, whereas the first remained above it.

Thus, the longer the HBc protein of which the VLP was formed, the more host cell residues were detected in the sample. The reasons remain uncertain, as other literature supports a similar trend for associated host nucleic acids, but a contradictory tendency for host cell proteins (Pusara et al., 2024; Sominskaya et al., 2013). Stability tests after purification showed that the capsid structure of the VLPs remained intact and correctly assembled over an extended period when stored in PBS containing 6 mM EDTA at 4 °C without obvious aggregation.

Taken together, we established a single-step chromatography purification method for HBc VLPs that can be broadly applied for different HBc variants, does not require specific lab equipment, and generates VLPs of high quality that can be stored at 4 °C.

## Data Availability

The original contributions presented in the study are included in the article/supplementary material, further inquiries can be directed to the corresponding author.
